# Case Report: A Case of Sintilimab-Induced Cystitis/Ureteritis and Review of Sintilimab-Related Adverse Events

**DOI:** 10.3389/fonc.2021.757069

**Published:** 2021-12-23

**Authors:** Lingfang Tu, Yuan Ye, Xiaoping Tang, Zhen Liang, Qihan You, Jianying Zhou, Zhijie Pan

**Affiliations:** ^1^ Department of Respiratory and Critical Care Medicine, The First Affiliated Hospital, College of Medicine, Zhejiang University, Hangzhou, China; ^2^ Department of Urology, The First Affiliated Hospital, College of Medicine, Zhejiang University, Hangzhou, China; ^3^ Department of Pathology, The First Affiliated Hospital, College of Medicine, Zhejiang University, Hangzhou, China

**Keywords:** immune checkpoint inhibitor, sintilimab, immune-related adverse events, cystitis, ureteritis

## Abstract

Immune checkpoint inhibitors (ICIs) have been proven to be beneficial in multiple advanced malignancies. However, the widespread use of ICIs also occurred with various immune-related adverse events (irAEs). Here, we first report a case of sintilimab-related cystitis/ureteritis. A 53-year-old man with driver gene-negative pulmonary adenocarcinoma (cT_1c_N_3_M_1c_, Stage IVB) was being treated with sintilimab in combination of paclitaxel-albumin and bevacizumab as second-line treatment. He was hospitalized for haematuria, pollakiuria, painful micturition and low back pain after three courses. Urinalysis showed red blood cells (RBCs) and white blood cells (WBCs) were obviously increased, and serum creatinine (sCr) level was also significantly elevated. Urine culture and cytology were both negative, and cystoscopy revealed diffused redness of bladder mucosa. Urinary ultrasonography showed mild hydronephrosis and dilated ureter. The patient was diagnosed as immunotherapy-related cystitis/ureteritis after a multidisciplinary team (MDT) meeting. Once the diagnosis was made, corticosteroid therapy was given, which rapidly resolved the patient’s symptoms and signs. Computer tomography angiography (CTA) and CT urography (CTU) was conducted after sCr level was back to normal and demonstrated ureter dilation and hydroureter. Once symptoms relieved, bladder biopsy was performed and confirmed the bladder inflammation. The patient was subsequently switched to maintenance dose of methylprednisolone and tapered gradually. Since sintilimab has been used in advanced malignancies, we first reported a rare case of sintilimab-induced cystitis/ureteritis and summarized sintilimab-related adverse events to improve the assessment and management of irAEs.

## Introduction

Cytotoxic T-lymphocytes-associated protein 4 (CTLA-4) and programmed cell death-1 (PD-1) are receptors that negatively regulate immune responses by inhibiting T-cell activation and proliferation (1). Immune checkpoint inhibitors (ICIs), which improve T-cell cytotoxicity against cancer *via* CTLA-4, PD-1 or PD-L1 blockade, are known to facilitate immunoactivity against malignancies by disinhibiting the anti-tumour responses of lymphocytes (2). As a member of ICIs, sintilimab (Tyvyt^®^) is a fully humanized immunoglobulin G4 monoclonal antibody that binding to PD-1, which in turn selectively blocks the interaction between PD-1 and its two known ligands, PD-L1 and PD-L2 (3). Sintilimab has shown efficacy in the treatment of multiple malignancies including lymphoma, lung cancer, hepatocellular carcinoma, and gastric cancer (4). Despite favourable outcomes, it could also lead to a wide spectrum of immune-related adverse events (irAEs) (5, 6). However, sintilimab-associated adverse events affecting the urinary tract have rarely been reported and discussed (5). Here, we first report a case of sintilimab-induced cystitis/ureteritis, and summarize sintilimab-related adverse events to achieve a better management and surveillance of irAEs.

## Case Presentation

**Figure 1:**
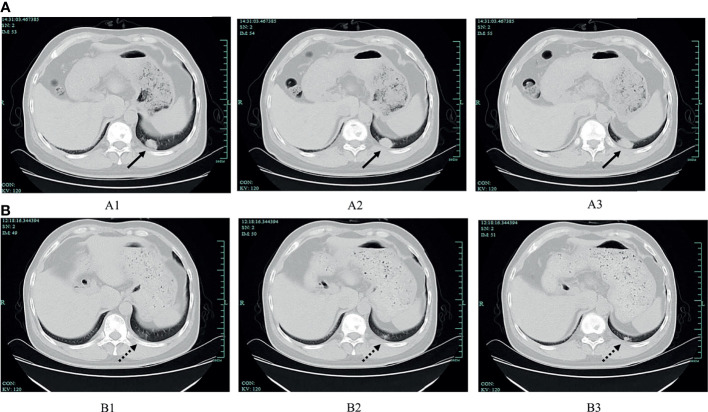
The comparison of chest CT before and after sintilimab treatment. **(A)** Chest CT scan before sintilimab treatment (July 2020). Images of A1, A2, and A3 consecutively showing the tumour at the posterior segment of the left lower lobe, and the tumour size was 2.1×1.4 cm, 2.4×1.5 cm, 2.7×1.5 cm, respectively (solid lines indicate the tumour). **(B)** Chest CT scan after sintilimab treatment (September 2020). Images of B1, B2 and B3 showing the tumour at the same location when compared with A1, A2, and A3, separately, and the tumour size was 0.4×0.4 cm, 0.5×0.4 cm, 1.0×0.7 cm, respectively (dotted lines indicate the tumour).

**Figure 2:**
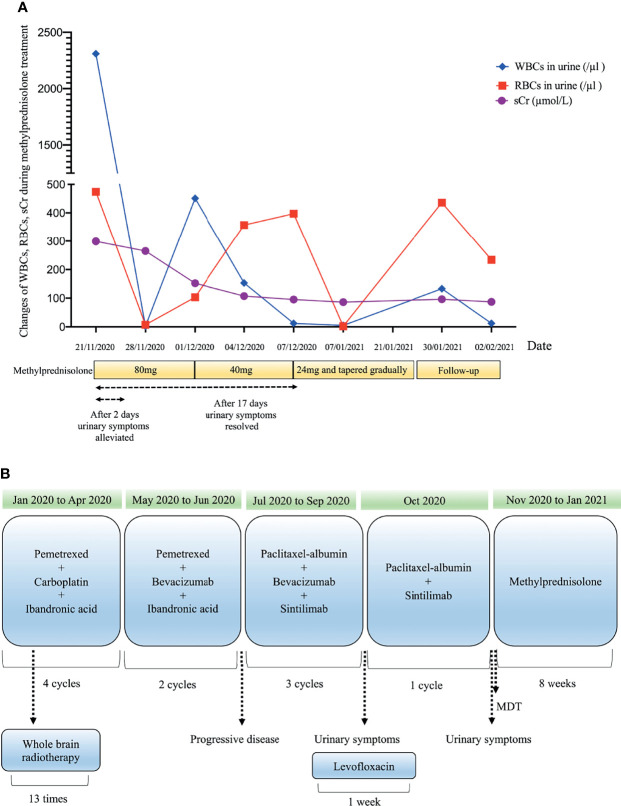
Clinical course over methylprednisolone therapy **(A)** and timeline of treatment course **(B)**. Drug dosage: bevacizumab, 7.5mg/kg; carboplatin, (area under the concentration-time curve) 4-5 mg/mL/min; ibandronic acid, 4mg; levofloxacin, 500mg; methylprednisolone (initial), 1mg/kg/d; paclitaxel-albumin, 260mg/m^2^; pemetrexed, 500mg/m^2^; sintilimab, 200mg.

**Figure 3:**
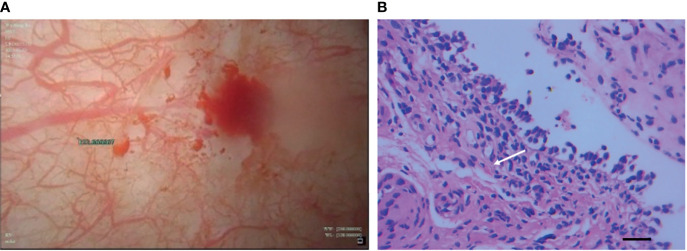
Cystoscopy of bladder **(A)** revealed diffuse redness of bladder mucosa and histopathological findings **(B)** of bladder biopsy showed lymphocyte-dominant bladder inflammation and interstitial tissue hyperplasia (white arrow), which was shown in the increased number of fibrocytes (H&E staining, magnification ×400, bar =25μm).

A 53-year-old man who had no smoking history was admitted to the first Affiliated Hospital of Zhejiang University (Hangzhou, China) with a mass in the posterior segment of the left lower lobe and complaint of upper right abdominal pain while without any respiratory symptoms. On physical examination, superficial right upper quadrant tenderness was noted. The patient was diagnosed with driver gene-negative pulmonary adenocarcinoma (cT_1c_N_3_M_1c_, Stage IVB) according to the eighth edition AJCC staging system. The PD-L1 tumour proportion score (TPS) was 50% using PD-L1 IHC 22C3 pharmDx (Dako North America, Inc., Carpinteria, CA, USA). The patient had a history of laparoscopic cholecystectomy due to gallstone and history of cefalexin allergy, and his family history of genetic diseases was negative. His performance score was 1. Initially, the patient received pemetrexed (Hansoh Pharmaceutical, Co., Ltd, Lianyungang, China) plus carboplatin (Corden Pharma Latina SpA, Sermoneta, Italy) for four cycles, followed by two cycles of pemetrexed and bevacizumab (Qilu Pharmaceutical Co., Ltd, Jinan, China). During first-line treatment, the best effect was assessed as partial response (PR), and the progression free survival (PFS) was about six months. Intravenous ibandronic acid (Institute of Pharmacy, Hebei Medical University, Shijiazhuang, China) was given to reduce metastatic bone pain throughout the first-line regular chemotherapy. Whole brain radiotherapy was conducted for thirteen times (3900 cGy in total) after the first cycle of first-line treatment. However, after six cycles of first-line chemotherapy, chest CT assessment of the patient demonstrated progressive disease according to Response Evaluation Criteria in Solid Tumors version 1.1 (RECIST v1.1) (7). The re-assessed staging of the patient was classified as rT1_c_N_3_M_1c_, stage IVB. The patient complained of alleviated upper right abdominal pain without other complaints, and physical examinations demonstrated mild right upper quadrant tenderness with no other alterations. Due to disease progression, he was administered sintilimab (200mg, Q21d, Innovent Biologics, Suzhou, China) in combination of paclitaxel-albumin (Hengrui, Pharmaceutical Co., Ltd, Lianyungang, China) and bevacizumab as second-line therapy. After two cycles of second-line treatment, response to treatment was assessed as PR (**Figure 1**). Urinalysis results were normal before sintilimab administration. Symptoms with haematuria, pollakiuria and painful micturition developed on the fifth day after the third course of second-line therapy, while no fever was observed. Urinalysis showed red blood cells (RBCs) of 3889.7/µl and white blood cells (WBCs) of 2133.5/µl. Urine culture and serum creatine (sCr) were normal. Urinary bladder ultrasound observed a thickened bladder wall and benign prostatic hyperplasia. Levofloxacin (Daiichi Sankyo (China) Holding Co., Ltd, Beijing, China) was administrated for seven days, which improved symptoms of bloody urine and urinary irritation. These symptoms were originally considered to be the adverse effect of bevacizumab; hence, bevacizumab was immediately discontinued. However, after the fourth cycle of paclitaxel-albumin and sintilimab treatment, similar urinary symptoms and low back pain occurred without fever. Urinalysis showed increased RBCs and WBCs. Urine culture and cytology were both negative. Blood tests showed WBCs of 10100/µl (neutrophils 66.5%, eosinophils 5.5%), C-reactive protein 52.1 mg/L, lactate dehydrogenase 166 U/L, and sCr 299 µmol/L. Urinary ultrasonography showed hydronephrosis and dilated ureter. Cystoscopy revealed diffuse redness of bladder mucosa. A multidisciplinary team (MDT) meeting was organized, and the diagnosis of sintilimab-induced cystitis/ureteritis (Grade 3) was made after exclusion the evidence of urinary infection and metastasis. Methylprednisolone (80mg, 1mg/kg/d, Pfizer Manufacturing Belgium NV, Puurs, Belgium) therapy was performed, and urinary symptoms of the patient was obviously alleviated after two days. The laboratory values were also decreased during corticosteroids treatment (**Figure 2A**). When sCr level was back to normal, computer tomography angiography (CTA)/CT urography (CTU) was conducted and demonstrated ureter dilation and hydroureter. Once urinary symptoms were alleviated, bladder biopsy was performed and confirmed the bladder lymphocyte-dominant inflammation and interstitial tissue hyperplasia (**Figure 3**). The patient was then switched to maintenance dose of methylprednisolone and tapered gradually, and the urinary symptoms were completely resolved after seventeen days of corticosteroid treatment. The whole steroid treatment was eight weeks. The timeline of treatment course was summarized in **Figure 2B**.

## Discussion and Review of the Literature

The wide use of ICIs shows significant benefits for patients with advanced malignancies, however, these agents could induce irAEs with nonspecific clinical symptoms and biomarkers (8). Although irAEs can affect any organ system, irAEs related to the urinary system have been rarely reported and discussed (9–13). Here, we first report an autoimmune cystitis/ureteritis caused by sintilimab.

IrAEs have been suggested to occur at any time, but usually develop within the first few weeks to months after administration initiation (8). In previous case reports of nivolumab-related cystitis, the time from initiation of therapy to development of cystitis ranged from two to twelve months (9–11, 13). In a case report of pembrolizumab-induced cystitis, urinary symptoms occurred after six cycles of treatment (12). In our report, the patient occurred with urinary symptoms after three cycles of sintilimab administration. Hence, it is necessary to aware the possibility of autoimmune cystitis for any newly onset urinary symptoms during ICIs therapy. The patient was diagnosed as autoimmune cystitis/ureteritis based on several reasons. First, the occurrence of urinary symptoms developed during sintilimab treatment, which raised the suspicion of irAEs. Second, there was no evidence of urinary infection and metastasis. Furthermore, the administration of corticosteroid obviously alleviated clinical symptoms and laboratory values of the patient.

PD-1 signalling permits self-tolerance in normal tissues, while tumour cells may hijack this signalling to evade host immune responses and establish a beneficial microenvironment for tumour growth and development (14, 15). Thus, PD-1 blockade could harm protective autoimmunity and lead to different types of irAEs *via* activated lymphocytes (15). The urinary bladder is immune-privileged because a number of chemicals, toxins and antigens constantly exposed to this site, therefore, it is essential to maintain the integrity and tolerance of the urothelium (16). The pathological features of a nivolumab-induced cystitis have shown epithelial desquamation and interstitial oedema (9). Although the precise mechanisms of ICIs-related cystitis/ureteritis are not fully elucidated, several studies have explored the potential mechanisms of immunotherapy-associated cystis. It has been demonstrated that lymphocytic infiltration, characterized by high CD8 and/or TIA-1 expression, was obviously increased in the urothelium of pembrolizumab-induced cystitis (12). This may indicate that some unknown antigens in the urothelium could be targeted by CD8^+^ and/or TIA-1^+^ lymphocytes, which consequently destroy the integrity of urothelial epithelum (12). PD-L1 knockout alone is not sufficient but is required to induce autoimmune cystitis in an animal model, indicating other mechanisms may get involved in the development of immune-cystitis (17). As to the PD-1 blockade will prevent the interaction of PD-1 and its known ligands PD-L1 as well as PD-L2 (3). PD-L2 is constitutively expressed by normal urothelium in healthy individuals and those with urothelial bladder cancer, suggesting a potential role in maintaining tolerance (16), whether PD-1/PD-L2 also participates in the autoimmune course is worthy to be explored.

In published clinical trials, sintilimab-related adverse events have been briefly documented (5). Here, we performed a detailed review of sintilimab-induced adverse events based on available case reports. For the cases reviewed, the demographic features, clinical manifestations and treatment of irAEs were summarized (**Table 1**). From these case reports, sintilimab has been shown to induce various immune-associated disorders, including autoimmune diabetes (18, 26, 30), pneumonitis (27, 28), pulmonary fibrosis (20), cytokine release syndrome and multiple organ failure (20, 21), myocarditis (24, 29), hypothyroid myopathy (19), myasthenia gravis and myasthenia crisis (22), paraneoplastic neurological syndrome and enteric neuropathy (23), psoriasis exacerbation (25). Some literatures previously indicated that patients with irAEs usually showed a markedly better efficacy than those without irAEs, known as higher overall response rate, longer PFS and better overall survival (31, 32). However, the relationship between new-onset autoimmune cystitis/ureteritis and the therapeutic response of PD-1 inhibitors is currently unknown.

**Table 1:** Sintilimab-related adverse events based on available case reports.

Diagnosis	Age, sex	Malignancies	Clinical presentations	Cycles of sintilimab	Treatment	Outcomes	Ref.
Autoimmune diabetes	56, male	HCC	Increased urination and drinking, increased fasting plasma glucose	Eight	Insulin therapy	Alive	Wen (2020) (18)
Hypothyroid myopathy	62, male	NSCLC	Elevation of creatine phosphokinase, fatigue	Four	Corticosteroids, levothyroxine	Alive	Ni (2020) (19)
Pulmonary fibrosis and cytokine release syndrome	50, male	Colon cancer	Fever, hypotension, shortness of breath	Two	Methylprednisolone and nintedanib	Alive	Hu (2020) (20)
Cytokine release syndrome, multiple organ injury	69, male	ESC	Fever, diarrhoea, leukopenia, renal damage	Three	Glucocorticoids and immunomodulators	Alive	Gao (2020) (21)
Myasthenia gravis overlap syndrome and myasthenia crisis, myocarditis	66, male	NSCLC	Fatigue, myalgia, render muscles	Two	Methylprednisolone, immunoglobulin, plasma exchange, mechanical ventilation, immunosuppressive therapy	Alive	Xing (2020) (22)
Paraneoplastic encephalitis and enteric neuropathy	66, female	SCLC	Focal seizure, speech disorder, intermittent amnesia, intestinal pseudo-obstruction	Two	Methylprednisolone, cefoperazone sulbactam, voriconazole	Alive	Kang (2020) (23)
Myocarditis	68, female	Breast cancer and Hodgkin’s lymphoma	Dyspnea, elevation of cardiac enzyme, abnormal ECG	One	Plasma exchange, tofacitinib and corticosteroids	Alive	Liu (2020) (24)
Psoriasis exacerbation	56, male	Lung adenocarcinoma	Red and swollen plaques, itching	One	Methylprednisolone	Alive	Sui (2020) (25)
Diabetic ketoacidosis	59, male	SCLC	Increased fasting glycemia level and HbA1c, positive urine ketone bodies, decreased blood pH and bicarbonate	Five	Insulin therapy	Alive	Huang (2021) (26)
Pneumonitis and hyperthermia	35, male	SCLC	Hyperthermia, wheezing and coughing, increased IL-6 level	One	Corticosteroids and tocilizumab	Alive	Li (2021) (27)
Pneumonitis	67, male	NSCLC	Dyspnea, rales of lung and low breath sound of the left thorax	Ten	Prednisolone	Alive	Dai (2021) (28)
Myocarditis	68, male	NSCLC	Productive cough and dysphagia, abnormal ECG, increased serum myocardial enzyme levels	Three	Methylprednisolone	Alive	Bi (2021) (29)
Autoimmune diabetes	42, male	HCC	Nausea, vomiting, thirst, polyuria and positive urine ketone body	Ten	Insulin therapy	Alive	Fu (2021) (30)

ESC, esophageal cancer; HCC, hepatocellular carcinoma; NSCLC, non-small cell lung cancer; SCLC, small cell lung cancer.

With regard to treatment of irAEs based on Common Terminology Criteria for Adverse Events (CTCAE), irAEs of Grade 2 or higher need to add temporary immunosuppressive agents such as oral glucocorticoids or additional immunomodulators (33). When steroids get involved in ICIs treatment, there is concern that its immunosuppressive effect could jeopardize the therapeutic effect of immunotherapy. However, the effects of steroids on ICIs are still controversial. In a systematic review, Garant et al. (34) demonstrated that the concomitant administration of steroids and ICIs may not result in worse outcomes and Kapoor et al. (35) also showed that the usage of steroid did not make a difference of the overall survival in patients with ICIs treatment. On the other hand, Arbour et al. (36) suggested that the usage of prednisone of more than 10 mg in advance of ICIs could negatively regulate the clinical effect of immunotherapy. The discrepancies between these studies may be ascribed to confounding variables in these studies, and well-designed prospective studies could be helpful to answer this question in the future. Once irAEs are well-controlled, it is reported that ICIs can be administrated again unless disease progression or other uncontrolled drug toxicities. However, sintilimab treatment was discontinued in this patient because of disease progression.

With the widespread use of ICIs in various cancer types, irAEs have raised lots of concerns in clinical practice. Early recognition and intervention of irAEs are crucial and recommended. Due to the complex mechanism and unspecific feature of irAEs, it always goes beyond the field of oncology and demands a collaborative and MDT approach to achieve favourable outcomes (37).

## Conclusion

Here, we report a case of nonbacterial cystitis/ureteritis associated with sintilimab and review sintilimab-related adverse events based on published case reports. As sintilimab alone or combined therapies are currently used in various malignancies, it is critical to be aware of its potential irAEs and to achieve adequate assessment and management.

## Data Availability Statement

The original contributions presented in the study are included in the article/supplementary material. Further inquiries can be directed to the corresponding authors.

## Ethics Statement

The studies involving human participants were reviewed and approved by Center for Ethics in the First Affiliated Hospital of Zhejiang University. The patients/participants provided their written informed consent to participate in this study. Written informed consent was obtained from the individual(s) for the publication of any potentially identifiable images or data included in this article.

## Author Contributions

LT designed the case report and drafted the manuscript. YY analyzed the patient data and revised the manuscript. XT made significant contributions to the collection of patient data. ZL provided significant contributions to the interpretation of CTA/CTU imaging. QY contributed to the analysis of pathological data. JZ and ZP provided significant contributions to the analysis of the patient data and designed the case report. All authors contributed to the article and approved the submitted version.

## Conflict of Interest

The authors declare that the research was conducted in the absence of any commercial or financial relationships that could be construed as a potential conflict of interest.

## Publisher’s Note

All claims expressed in this article are solely those of the authors and do not necessarily represent those of their affiliated organizations, or those of the publisher, the editors and the reviewers. Any product that may be evaluated in this article, or claim that may be made by its manufacturer, is not guaranteed or endorsed by the publisher.

## Abbreviations

CTA, computer tomography angiography; CTCAE, common terminology criteria for adverse events; CTLA-4, cytotoxic T-lymphocytes-associated protein 4; CTU, computer tomography urography; ICIs, immune checkpoint inhibitors; irAEs, immune-related adverse events; MDT, multi-disciplinary team; PD-1, programmed cell death-1; PFS, progression free survival; PR, partial response; RBCs, red blood cells; TPS, tumour proportion score; WBCs, white blood cells; sCr, serum creatinine.
